# Group, Blended and Individual, Unguided Online Delivery of Mindfulness-Based Cognitive Therapy for People With Cancer: Feasibility Uncontrolled Trial

**DOI:** 10.2196/52338

**Published:** 2024-02-21

**Authors:** Nasim Badaghi, Mette van Kruijsbergen, Anne Speckens, Joëlle Vilé, Judith Prins, Saskia Kelders, Linda Kwakkenbos

**Affiliations:** 1 Department of Psychiatry Radboud University Medical Center Nijmegen Netherlands; 2 Department of Medical Psychology Radboud University Medical Center Nijmegen Netherlands; 3 Department of Psychology, Health, and Technology University of Twente Enschede Netherlands; 4 Optentia Research Unit North West University Vanderbijlpark South Africa; 5 Department of IQ Healthcare Radboud University Medical Center Nijmegen Netherlands; 6 Department of Clinical Psychology Behavioural Science Institute Radboud University Nijmegen Netherlands

**Keywords:** cancer, eHeath, online interventions, mindfulness, psycho-oncology, qualitative research, oncology, CBT, blended, eMBCT, iCBT, cognitive therapy, unguided, psychotherapy, MBCT, co-creation, therapist, self-guided, peer-support, co-design, participatory

## Abstract

**Background:**

Online mindfulness based cognitive therapy (eMBCT) has been shown to reduce psychological distress in people with cancer. However, this population has reported lack of support and asynchronous communication as barriers to eMBCT, resulting in higher nonadherence rates than with face-to-face MBCT. Using a co-creation process, we developed 2 formats of eMBCT: group, blended (combination of therapist-guided group and individual online sessions) and individual, unguided (individual, unguided online sessions only). Group, blended eMBCT offers peer support and guidance, whereas individual, unguided eMBCT offers flexibility and the possibility of large-scale implementation.

**Objective:**

The objective of this nonrandomized feasibility study was to assess aspects of feasibility of the group, blended and individual, unguided eMBCT interventions.

**Methods:**

Participants were people with cancer who chose between group, blended and individual, unguided eMBCT. Both intervention conditions followed the same 8-week eMBCT program, including an introductory session and a silent day (10 sessions total). All sessions for individual, unguided eMBCT occurred via the platform Minddistrict, whereas group, blended eMBCT consisted of 3 online videoconference sessions guided by a mindfulness teacher and 5 sessions via Minddistrict. We assessed the feasibility of the intervention quantitatively and qualitatively by evaluating its acceptability among participants. Additionally, we assessed limited efficacy by looking at the number of questionnaires participants completed pre- and postintervention.

**Results:**

We included 12 participants for each eMBCT condition. Participants in group, blended eMBCT completed, on average, 9.7 of 10 sessions, compared with an average 8.3 sessions for individual, unguided eMBCT (excluding dropouts). Of the 24 participants, 13 (54%) agreed to be interviewed (5 unguided and 8 blended). Participants in both conditions reported positive experiences, including the convenience of not having to travel and the flexibility to choose when and where to participate. However, among the barriers for participation, participants in the group, blended condition reported a preference for more group sessions, and participants in the individual, unguided condition reported a lack of guidance. Additionally, for the group, blended condition, the effect sizes were small for all outcome measures (Hedges *g* range=0.01-0.36), except for fatigue, which had a moderate effect size (Hedges *g*=0.57). For the individual, unguided condition, the effect sizes were small for all outcome measures (Hedges *g* range=0.24-0.46), except for mindfulness skills (Hedges *g*=0.52) and engagement with the intervention (Hedges *g*=1.53).

**Conclusions:**

Participants in this study had a positive experience with group, blended and individual, unguided eMBCT. Based on the results from this study, we will adjust the intervention prior to conducting a full-scale randomized controlled trial to evaluate effectiveness; we will add 1 group session to the group, blended eMBCT using Zoom as the platform for the group sessions; and we will send reminders to participants to complete questionnaires.

**Trial Registration:**

ClinicalTrials.gov NCT05336916; https://clinicaltrials.gov/ct2/show/NCT05336916

## Introduction

The number of people with cancer is increasing at alarming rates. It has been estimated that, by 2040, the number of people with cancer will be almost double that of 2020 [1]. Additionally, approximately 1 in 3 individuals with cancer experiences severe psychological distress [2]. As a result, there is an increasing number of distressed people with cancer who could benefit from effective psycho-oncological interventions [3].

One kind of evidence-based psychological treatment for people with cancer is a mindfulness-based intervention (MBI). Mindfulness can be defined as moment-to-moment awareness, which is cultivated by purposely paying attention to the present experience without judgment [4]. Although mindfulness practices were originally developed centuries ago in the Buddhist traditions of Asia, it was not until the past couple of decades that they were implemented in health care. Different forms of MBIs (eg, mindfulness-based cognitive therapy [MBCT]) have been used across conditions [5], including cancer [6-8], and have been shown to have beneficial effects on psychological distress, quality of life, and well-being [5]. MBCT includes mindfulness components (eg, meditations, visualization exercises, movement exercises) and cognitive components from cognitive behavioral therapy (eg, identifying and reframing automatic thoughts, recognition that thoughts are not facts, habitual thoughts and behavioral patterns).

MBIs have been successfully adapted to online formats [9]. Although eHealth interventions are complex and relatively new [10], online MBIs offer multiple advantages over face-to-face interventions. For instance, online interventions are more easily accessible, more flexible in when and how participants can follow the program, and less costly [9]. A recent systematic review evaluated 9 randomized controlled trials (RCTs) and found that, although online MBIs generally have smaller effect sizes than face-to-face MBIs, they were still effective in reducing depression symptoms, anxiety, and stress, as well as improving mindfulness skills among people across different physical conditions [11]. In addition, a meta-analysis evaluated the effectiveness of different forms of online MBIs (delivered on a website or by an application) for people with cancer and found that online MBIs were effective in reducing distress, depression, and sleep disturbance and that they improved quality of life [12]. Plus, the authors of this meta-analysis concluded that online MBIs may provide unique advantage of increased accessibility and scalability. Online MBIs can offer a valuable alternative to face-to-face interventions, in particular for people with cancer who often already have to deal with frequent hospital visits, physical symptoms from the disease, and its treatment (such as intensive medical treatments and treatment-related fatigue and pain) [13].

Our research group previously conducted an RCT comparing the effectiveness and cost-effectiveness of online mindfulness-based cognitive therapy (eMBCT) and face-to-face MBCT with treatment as usual in reducing psychological distress in people with cancer (BeMind trial) [13]. The online condition in the BeMind trial consisted of an individual, 8-week, online mindfulness intervention supported by a qualified mindfulness teacher who provided feedback via email. The face-to-face condition was a prototypical 8-week group MBCT taught by a qualified mindfulness teacher.

Results from the BeMind trial showed that, in a heterogeneous sample of distressed people with cancer, both interventions were more effective at reducing psychological distress and were less costly than treatment as usual [13]. Nevertheless, nonadherence rates were higher in the individual eMBCT condition than in the group, face-to-face MBCT condition. Furthermore, qualitative analyses showed important barriers to participating in eMBCT, including insufficient peer support and asynchronous communication [14]. Additionally, mindfulness teachers had to invest more time for the individual, online condition than for the group condition, which may hamper large-scale implementation. Thus, the BeMind study showed that, although eMBCT is effective at reducing distress in people with cancer, there is room to improve the eMBCT intervention prior to implementation.

Considering the results from the BeMind trial and the social restrictions from the COVID-19 pandemic at the start of our project, we developed 2 new eHealth formats using a cocreation process. With experts in eHealth interventions, MBCT teachers, representatives from cancer patient organizations, and people with cancer, we explored how to give proper counseling, personalize the intervention, and make it more engaging. In addition, as adherence to online interventions without the guidance of a teacher (individual, unguided interventions) is often lower than intended, persuasive technology known to improve adherence, such as reminders and virtual coaches, was included [15]. By considering the different perspectives of the stakeholders, we aimed to develop a more appealing, persuasive, and participant-focused online intervention.

We developed the following 2 interventions using a cocreation process: group, blended and individual, unguided eMBCT. Group, blended eMBCT consisted of 3 online, group sessions with a mindfulness teacher and 5 individual, teacher-assisted online sessions; this combination provided peer support and partly synchronous communication. Individual, unguided eMBCT consisted of 8 eMBCT sessions in which participants followed the intervention by themselves without teacher guidance; an unguided intervention could increase access and improve scalability at a lower cost for both participants and therapists. Both intervention conditions also included an introductory session and a silent day. We developed group, blended and individual, unguided interventions to optimize eMBCT delivery and efficacy by addressing the barriers we found in our previous study, by considering the target group needs and by constructing an online intervention that is engaging and attractive.

Although the 2 MBIs were carefully designed using a cocreation process with relevant stakeholders, aspects of their feasibility such as acceptability and preliminary efficacy needed to be established prior to conducting a full-scale RCT. In fact, pilot studies can support researchers with identifying possible challenges, weighing resources, and evaluating the feasibility of an intervention [16,17]. Moreover, pilot studies can assess preliminary efficacy of an intervention before moving on to a full-scale RCT, which involves more resources [16,17]. The objective of this pilot study was to assess the feasibility and preliminary efficacy of the group, blended and individual, unguided eMBCT interventions among people with cancer. The results from this pilot will help us improve the intervention conditions prior to testing their effectiveness in a full-scale, 3-arm RCT that will compare group, blended and individual, unguided eMBCT with care as usual [18].

## Methods

### Study Design and Setting

This was a mixed methods, nonrandomized feasibility study. Participants could choose to participate in either group, blended or individual, unguided eMBCT. All participants were invited to a semistructured interview postintervention. Participants were also asked to complete questionnaires before and after the intervention.

The study was conducted at the Radboudumc Center for Mindfulness in Nijmegen, The Netherlands. Although our study was not randomized, results are reported in accordance with the Consolidated Standards of Reporting Trials (CONSORT) extension for randomized pilot and feasibility trials [19], as many of the principles described apply.

### Participants

Participants were eligible if they (1) were adults and had been diagnosed with cancer at any point in their life (irrespective of type or stage of cancer and time since diagnosis); (2) had internet access and were able to use a computer; and (3) had good command of the Dutch language. Participants were excluded if they (1) had participated in a mindfulness intervention before (>4 sessions); (2) had a severe psychiatric comorbidity that warranted acute treatment (ie, psychosis, mania, severe personality disorders, suicidal thoughts); (3) had dependence on drugs or alcohol; or (4) had severe cognitive impairments.

### Procedure

Participants were recruited online via posts placed on websites of cancer-related organizations or patient group forums (online sites where people with cancer can hold conversations and find information), flyers and posters placed at Radboud University medical center, and social media platforms.

Interested participants contacted us via email, via phone, or by completing the contact form on the study website. After this, they received a phone call from one of the researchers to verify inclusion criteria and provide information about the study. Participants were allowed to choose their preferred intervention condition. For each eMBCT condition, a maximum of 12 participants were allowed, so once one eMBCT condition was full, participants were informed that they could only participate in the other one if they wished to be included in this study. Eligible participants were sent the written information about the study and the informed consent form by post and email. After participants signed and returned the informed consent form, they were enrolled in the study and asked to complete pre-intervention questionnaires via the secure Castor EDC system.

Within 1 week after the 8 weeks of the intervention, participants were invited to complete postintervention questionnaires and share their experiences in a semistructured interview. Participants in both conditions were allowed to have any form of medical, psychological (except for MBIs), or paramedical care they required during the study period.

**Figure 1:**
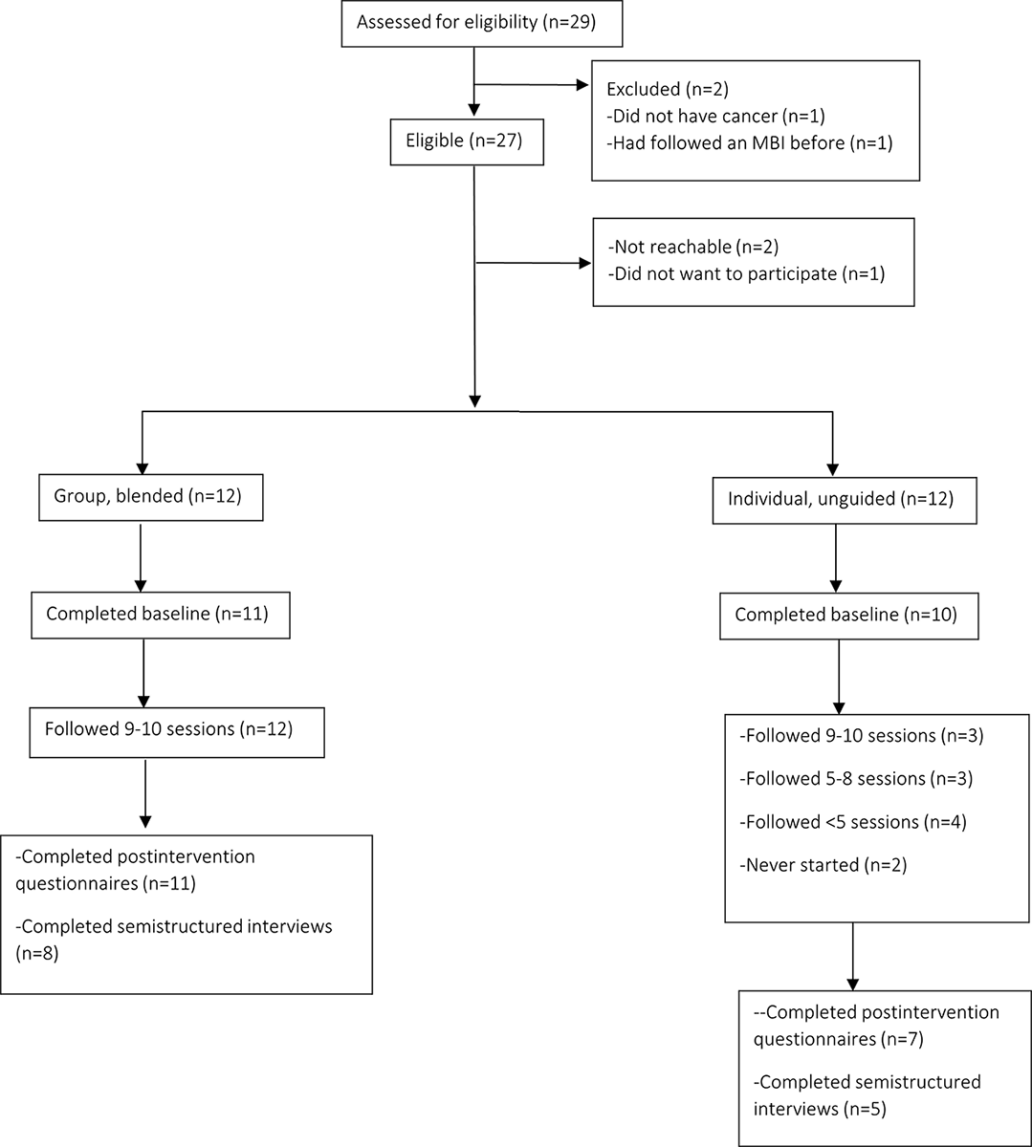
Participant inclusion flow chart, from eligibility assessment to study completion. MBI: mindfulness-based intervention.

See Figure 1 for the participant inclusion flow chart.

### Intervention

#### eMBCT

The content of both eMBCT interventions was based on the MBCT program developed by Segal et al [20]. For both conditions, the intervention consisted of 8 online sessions with mindfulness meditation exercises, psychoeducation, and reflections, plus a silent day. We included psychoeducation about mindfulness for cancer and grief and adapted the moving exercises for people with cancer. Participants were asked to do home practice for 30 minutes to 45 minutes a day. Although the content of the sessions did not differ between the 2 conditions, the delivery format did; in the group, blended eMBCT, sessions 1, 5, and 8 took place as online group sessions via the videoconferencing platform Zaurus. In both conditions, participants were allowed to join with a significant other. The specific content for each session has been published elsewhere [18].

##### Group, Blended eMBCT

Group, blended eMBCT consisted of 3 videoconference group sessions lasting 2.5 hours and guided by a mindfulness teacher (sessions 1, 5, and 8). The other sessions (sessions 2, 3, 4, 6, and 7 and the silent day) were followed individually via Minddistrict. Participants were provided with written online feedback on the individual sessions from their mindfulness teacher within Minddistrict. The mindfulness teachers involved in the project were health care professionals experienced in psycho-oncology who met the qualification criteria of the Association of Mindfulness Teachers based in The Netherlands and Flanders, which are in line with the 2015 UK Network for Mindfulness-Based Teachers criteria. In addition, teachers had regular peer supervision sessions led by a senior mindfulness teacher (AS).

##### Individual, Unguided eMBCT

Participants in the unguided eMBCT condition were provided the entire training through Minddistrict. They received weekly access to one of the online mindfulness sessions, which involved the same themes, exercises, and homework as those in the group, blended eMBCT condition. However, there was no mindfulness teacher involved. Participants received automated feedback instead. Participants could contact the research team for technical support.

### Feasibility Outcomes

Aspects of feasibility were assessed based on the areas of focus suggested by Bowen et al [21], including acceptability and limited efficacy. Acceptability focuses on how the participants react to the intervention (to what extent the intervention is suitable, satisfying, and attractive) [21]. Limited efficacy intends to test the intervention in a limited way [21]. For this pilot study, limited efficacy was evaluated with the pre-intervention and postintervention questionnaire scores (the same questionnaires will be used in the full-scale RCT).

*Acceptability* of the intervention was evaluated by how many participants chose each eMBCT condition, how many participants started the intervention, the participants’ clinical characteristics at baseline, the average number of sessions completed per condition—adherence—(through attendance lists for groups and login data in Minddistrict), and dropout rate (participants who discontinued the intervention).

In addition, acceptability was evaluated by conducting semistructured interviews postintervention. Participants from group, blended and individual, unguided eMBCT were asked program-specific questions to assess their experiences with the respective intervention condition; in addition, they were asked to express if they experienced barriers to or facilitators for group, blended and individual, unguided eMBCT (eg, Were there specific parts that were helpful/not helpful?). Questions were asked in an open way to permit participants to freely speak. Participants were interviewed via telephone within 3 months after the end of the intervention. Participant interviews were conducted by 2 researchers with previous experience in qualitative research who had not been involved in the delivery of the training (see Multimedia Appendix 1 for the complete interview guide).

*Limited efficacy* includes planned outcome measures for full-scale RCT. Consistent with the feasibility trial design [19,22], limited efficacy of the following measures of distress and secondary outcomes that will be used to evaluate the program in the full-scale trial are reported: Hospital Anxiety and Depression Scale [23], severity scale of the Fear of Cancer Recurrence Inventory-Short Form [24,25], fatigue severity subscale of the Checklist Individual Strength [26], rumination subscale of the Rumination and Reflection Questionnaire [27], Five Facet Mindfulness Questionnaire-Short Form [28], Self-Compassion Scale-Short Form [29], Mental Health Continuum-Short Form [30], Twente Engagement with E-health Technologies Scale [31]. Detailed information about each questionnaire has been published previously [18].

### Sample Size

There is no consensus about the optimal sample size for pilot and feasibility studies [32]. Guidance varies between 12 and 30 participants or more per trial arm [33,34]. To reach the study objectives, including sufficient feedback on the acceptability of both conditions, we aimed to include 12 participants in the individual, unguided condition and 12 participants in the group, blended condition.

### Data Analyses

Descriptive statistics were used to characterize participant demographics, recruitment numbers, and login data. Baseline and postintervention mean scores, standard deviations, and effect sizes from all the questionnaires were calculated for both conditions using SPSS (version 27; IBM Corp). The number of sessions completed was calculated by summing the number of sessions that each participant completed. In this count, we included the introductory session and the silent day, which made a total of 10 sessions. The proportion of sessions completed in Minddistrict was represented as percentages based on each participant’s usage login data. We transformed the percentages into units to aggregate the number of sessions completed by each participant.

Interviews were transcribed verbatim and analyzed by means of an iterative process of thematic analysis in which coding categories were derived directly from the text data (inductive coding) [35]. We followed a form of data-driven thematic analysis and followed the different phases of thematic analysis as suggested by Braun and Clark [35]. First, we familiarized ourselves with the data. Second, 2 researchers independently did a first round of coding 2 interviews (1 from a participant in the group, blended intervention and 1 from a participant in the individual, unguided intervention), during which initial codes were generated. Third, codes between the 2 researchers were compared and reviewed. Fourth, the 2 researchers created a common coding map for analysis and coded the rest of the interviews. All remaining interviews were coded by one researcher and reviewed by a second. Discrepancies were discussed and resolved by consensus. Fifth, the codes from step 4 were reviewed and categorized into broader themes with a larger group. The group consisted of 3 senior researchers (AS, JP, and LK) who have extensive experience in the fields of mindfulness, cancer, and research methodology, a mindfulness teacher with more than 20 years of experience teaching different mindfulness courses (including mindfulness for people with cancer), and a PhD candidate with a background in mindfulness and clinical experience (NB). Definitions and labels for each theme and subthemes were generated. Finally, we selected vivid and compelling examples from the interviews that clearly portrayed the themes and subthemes identified.

### Ethical Considerations

The Buddy feasibility trial was approved by the ethical review board, CMO Arnhem-Nijmegen (number: NL73117.091.20), prior to data collection. The study was conducted according to the principles of the Declaration of Helsinki (6th edition, 2008) and in accordance with the Medical Research Involving Human Subjects Act (WMO). All participants who joined the study signed an informed consent form prior to enrollment. Participation was free of charge and voluntary. Participants were informed that they could withdraw at any time without consequences, that their anonymity was ensured, and that there was no monetary compensation for participation.

## Results

### Acceptability

Enrollment took place between August 2020 and January 2021. A total of 29 participants were assessed for eligibility, of which 2 were excluded; 1 participant had previously followed an MBI, and the other participant was never diagnosed with cancer. Of the 27 eligible participants, 1 participant did not want to participate, 1 participant preferred to join a group, face-to-face MBI program, and we could not contact the other person. In total, 24 participants started the program. The first 12 eligible participants who contacted us preferred to join the group, blended condition. Therefore, subsequent eligible applicants were only offered individual, unguided eMBCT*.* See Figure 1 for the complete flow of participant selection procedures.

All participants were Dutch and had at least a secondary education; their mean age was 51 (SD 11.5) years, and most (21/24, 87%) were female. Participants had the following types of cancer: breast (9/21, 42%), ovarian (5/21, 24%), colon (3/21, 14%), leukemia (2/21 ,10%), lymphoma (1/21 ,5%), and bowel (1/21, 5%). Most participants (15/20, 75%) received treatment with curative intent. In addition, overall, it appeared that participants in the blended group had more working hours than participants in the unguided group (21 hours vs 13 hours) and were more often treated with curative intent (10/11, 90% vs 5/9, 56%), and a larger proportion was diagnosed with ovarian cancer (5/11, 46% vs 0/10, 0%). Demographic and disease characteristics of the participants for each condition are shown in Table 1.

For the group, blended condition, the mean number of sessions completed was 9.7 of 10; all participants completed at least 9 of the 10 sessions (minimum 9 sessions and maximum 10 sessions); adherence was high; and there were no dropouts.

In total, 5 of the 12 (42%) participants in the unguided, online condition dropped out. These participants completed fewer than 4 sessions: 3 participants completed 3 sessions, and 2 participants completed 1 session only. We could not contact 2 participants after they dropped out, and the other 3 reported that the program was too hard to follow because of personal circumstances. Participants in the unguided, individual condition who dropped out and completed baseline assessments (n=4) had metastatic cancer and were receiving palliative anticancer treatment. The mean number of sessions completed for the individual, unguided condition, excluding dropouts, was 8.3 of 10 (minimum 1.5 sessions and maximum 10 sessions). The mean number of sessions completed for the individual, unguided condition, including dropouts, was 6.1 of 10.

**Table 1:** Demographic and clinical characteristics of the included participants at baseline.

Characteristics			Blended (n=12)		Unguided (n=12)
Age (years), mean (SD)			51 (2.3)		51 (4.4)^a^
Female sex, n (%)			11 (92)		10 (83)
Married or living as married, n (%)			9 (82)^a^		9 (90)
Dutch nationality, n (%)			12 (100)		12 (100)
Work per week (hours), mean (SD)			21 (11)^b^		13 (14)^c^
**Education, n (%)**					
	Secondary	6 (54)^a^		4 (40)^d^	
	Tertiary	5 (45)^a^		6 (60)^d^	
**Anticancer treatment, n (%)**					
	Curative	10 (90)^a^		5 (56)^d^	
	Palliative	1 (10)^a^		4 (44)^d^	
Duration since first cancer diagnosis (months), mean (SD)			14 (14)^a^		18 (24)^b^
**Type of cancer, n (%)**					
	Breast	3 (27)^a^		6 (60)^b^	
	Ovarian	5 (46)^a^		0^b^	
	Colon	1 (9)^a^		2 (20)^b^	
	Acute myeloid leukemia	0^a^		2 (20)^b^	
	Lymphoma	1 (9)^a^		0^b^	
	Bowel	1 (9)^a^		0^b^	
Previous experience with meditation (yes), n (%)			6 (55)^a^		4 (40)^b^

^a^n=11.

^b^n=10.

^c^n=8.

^d^n=9.

### Results From the Interviews

#### Participation and Themes

All 24 participants were invited for semistructured interviews, and 13 (54%) agreed to participate (5 from the individual, unguided condition and 8 from the group, blended eMBCT condition). Interviews lasted between 35 minutes and 90 minutes. Responses from the semistructured interviews showed that there were overlapping as well as different barriers and facilitators for group, blended and individual, unguided eMBCT. The barriers and facilitators were organized into the following 4 emergent themes (each one with multiple subthemes): program content; program format; group, blended condition; and individual, unguided condition.

#### Program Content

Factors categorized as program content were the specific online program components (eg, exercises, videos, diaries) in Minddistrict that were used in both intervention conditions. Program content facilitators that participants reported included the possibility to choose different exercises that were suitable to personal needs. For instance, one participant reported:

What I also like is that you could choose which exercises yourself (...) that's just really nice, that bit of freedom you had.

group, blended condition; completed 10 sessions

In addition, participants reported that the identification with other peer participants in the videos and the normalization of their experiences through video stories were useful and made them feel less alone:

I really liked those videos, to experience what other people thought about it—how they thought about it or how they experienced it (…) I often recognized myself in it, so that was helpful.

individual, unguided condition; completed 7 sessions

Reported program content barriers included too many exercises with no clear explanation about their rationale, too many forms to fill out, and reflection on emotions in diaries that were challenging and confrontational; for instance, 1 participant reported the following:

It was sometimes quite intense to fill in your diary every day, and every week there was also another diary that you had to keep.

group, blended condition; completed 10 sessions

#### Program Format

Factors categorized as program format were the arrangement of the online program that facilitated participants’ participation (eg, structure, time, place) to both intervention conditions. Not having to travel and being able to follow the program at one’s own pace were the most reported program format facilitators. One participant reported:

I liked doing it at home so I could do it in my own time and place, and also with no travelling times.

individual, unguided condition; completed 10 sessions

Additionally, the presentation of visual information that complemented the written and spoken exercises and having the choice of a physical booklet as an additional source and future reference were positively valued, as participants indicated:

I always really liked the information pieces with those drawings, because then I could make it visual instead of everything being spoken, then I really had an image and I really liked that.

individual, unguided condition; completed 7 sessions

...a book you can easily pick up in addition to your exercise. I personally prefer it.

individual, unguided condition; completed 10 sessions

However, some other participants said that they found the program structure unclear and that it was difficult to navigate. In addition, they reported that it required too much time investment and that it was difficult to follow it at home with constant interruptions of family members. For instance, one participant from the individual, unguided condition said:

I just don't know when to schedule it. Then, I had just found a moment, and another child came downstairs and asked loudly: ‘Can I have an apple?’ Yes. Or then, the partner comes and gets some tea, and he would say… ‘Oh, sorry, I see you are doing mindfulness.’

individual, unguided condition; completed 7 sessions

#### Group, Blended Condition and Individual, Unguided Condition

There were also barriers and facilitators that were specific to the intervention conditions. Participants in the group, blended condition liked the group sessions because they had connection with others, peer support, the possibility to ask questions, and synchronicity in communication. A participant in the group, blended condition who completed 10 sessions reported that “it's also nice to hear that other people are struggling with the same things and yes, you know, you're suddenly not crazy anymore.”

Another participant reported that:

I was really looking forward to it when it was finally time for another group session of, oh yes, nice, just talking to people.

group, blended condition; completed 10 sessions

Moreover, one participant emphasized that:

You know, if you have any questions, at least you can ask questions. Then, you will get an answer right away.

group, blended condition; completed 9 sessions

Barriers that were specific to the group, blended sessions included having to be sitting for a long time during the group sessions, the intensity and length of the sessions, the infrequent number of group sessions, and the fact that they were online rather than in person. A participant commented that “and with the 3 times you only have together, that was quite short” [group, blended condition; completed 9 sessions]. Another participant, also from the group, blended condition, reported that “it's quite a long time to sit behind a screen like that. And I wasn't that far into my recovery yet, so I especially thought the first session was really exhausting” [completed 10 sessions]. An outstanding result was that almost all participants from the group, blended condition indicated the need to have more group sessions. They mentioned that the group sessions were not enough to get to know each other properly. One participant reported:

I was really looking forward to when it was finally time again for a group session (...) I think for me, if it had been a group session 8 times, then that would also just be very nice.

group, blended condition; completed 10 sessions

Participants in the individual, unguided condition reported lack of peer support and lack of feedback from a therapist as barriers for participation. One participant from the individual, unguided condition reported that “the fact that there is no contact with a person or with a group and that there is also no concrete agreement that we will meet each other—even if it is only online...that made it very difficult for me to keep it up” [completed 6 sessions].

For all the barriers and facilitators for both conditions across themes and subthemes, see Table 2.

**Table 2:** Barriers and facilitators experienced by participants during group, blended and individual, unguided online mindfulness-based cognitive therapy (eMBCT).

Themes and subthemes		Facilitators	Barriers
**(1) Program: content^a^**			
	Exercises	Multiple options: different exercises and different voices for the meditations Pleasant and clear voices that become familiar with time Good and short exercises	Too many exercises. No instructions explaining the goal of the meditation exercises
	Silent day	Pleasant silent day	Not feasible to do it independently at home without distractions Difficult to separate from daily disturbances and quotidian environment Too long silent day Lack of peer support and guidance from a therapist
	Diaries	Promotion of personal reflection Support personal processes	Too many different forms to fill out every week Confronting to fill out a diary every day
	Automatic feedback	Recognition with peer participants and normalization	Too impersonal Participants forced to choose an answer before being able to proceed
	Videos	Encouraging to see that the program is helpful for other people with cancer Explanations that clarify what is meant by the elements of the program Relate to other people with cancer experiences	Set too high standards Videos not inclusive enough
	Reminders	Helpful reminders	Too many reminders
**(2) Program: format^a^**			
	Initial contact research team	Very helpful to have a personal introduction into the online program, makes it accessible	Business-like communication style Too impersonal
	Help desk	Supportive if you ran into problems Helpful to have the option of personal contact	Sometimes, it took too long to respond to participants.
	Structure	Logical structure: the sessions build on each other consistently. Lot of suggestions Clear structure of the platform	Unclear where to write notes or not write them at all Unclear structure, repetition; what do I need to do?
	Navigating through program	Easy to move forward in the program Possible to look back at own notes Did not get stuck Being able to fill things out yourself	Navigating the program was difficult. Not clear how to save entered information Unclear where to put notes in both daily and weekly forms Not being able to go back to the exercises to do them again or to the diaries to add information later on Getting stuck, not being able to move forward
	Time	Very relaxed, own time, own planning No travelling time Possibility to combine eMBCT with cancer treatment, rehabilitation, household chores Option to adapt the time invested in the program to the energy levels	Time-consuming program, took too much time
	Place	Pleasant to do it at home No traveling, does not cost energy	Interruptions for family members Difficult to find a room in the house where you will not be disturbed
	Infographics and avatar	Possibility to choose the coach and answers	The avatar was not of any added value for some participants. Getting stuck if an avatar was not selected
	Physical booklet	Having the choice of a physical booklet Being able to look back in the physical booklet to previous sessions, besides the online program Having an additional source for reading Future reference	Too many different things: online program, booklet; unsure where to go For some people, the app was very clear, and they used the app only. There was no added value from the booklet.
**(3) Group, blended condition**			
	Group sessions	Connection with others Peer support Possibility to ask questions Synchronicity in communication Recognition that others struggle with the same things	Being stressed about not being able to log in in time Not being able to see people properly in the screen (Zaurus), no speaker perspective Prefer to meet people in person rather than on a screen Very tiring to sit behind a screen for a long time Intense, long, and tiring group sessions Too infrequent Confrontation with other participants’ cancer
	Feedback from mindfulness teacher	Good quality, elaborated, and personalized feedback Trustworthy, accessible, and supportive	Even though people got written feedback, this was less stimulating to some. Asynchronous written feedback and not clear timing of receiving it
**(4) Individual, unguided condition**			
	Lack of peer support	—^b^	Need for self-discipline Difficult to maintain engagement without appointments Lack of support from a community
	Lack of feedback therapist	—	Feels unsafe to share personal information with unknown recipient

^a^The themes for the program content and format and their respective subthemes applied to both intervention conditions.

^b^No response.

### Planned Trial Outcomes

Overall, 21 of the 24 (88%) participants completed baseline questionnaires, and 19 of the 24 (79%) participants completed posttreatment questionnaires. More specifically, most participants (11/12, 92%) in the group, blended condition completed both baseline and posttreatment questionnaires, while in the individual, unguided condition, only 7 (7/12, 58%) completed both baseline and posttreatment questionnaires. Although this study had a small sample size and tests of significance were not included, effect sizes were calculated to evaluate changes between pre- and postassessments. For the group, blended condition, the effect sizes for change before and after treatment were small for all outcome measures (Hedges *g* range=0.01-0.36), except for fatigue, which had a moderate effect size (Hedges *g*=0.57). For the individual, unguided condition, the effect sizes for change before and after treatment were small for all outcome measures (Hedges *g* range=0.24-0.46), except for mindfulness skills (Hedges *g*=0.52) and engagement with the intervention (Hedges *g*=1.53). Table 3 shows the baseline and postintervention scores for the planned trial outcome measures for both groups. No adverse events were reported.

**Table 3:** Baseline and postintervention scores after the 8-week intervention for the planned Buddy trial outcome measures.

Measurements	Score in the blended group (n=12), mean (SD)			Effect size, Hedges *g* (95% CI)	Score in the unguided group (n=12), mean (SD)			Effect size, Hedges *g* (95% CI)
	Baseline	Postintervention			Baseline	Postintervention		
Psychological distress (HADS^a^)	13.6 (7.1)^b^	12.1 (6.8)	0.21 (–0.61 to 1.03)		18.4 (8.3)^c^	17.4 (5.8)^d^	0.13 (–0.84 to 1.10)	
Fear of cancer recurrence (FCRI-SF^e^)	78.4 (18.6)^b^	78.5 (14.2)	0.01 (–0.82 to 0.81)		94.7 (18.5)^c^	93.1 (15.1)^d^	0.09 (–0.88 to 1.05)	
Fatigue (CIS^f^)	33.7 (5.5)^b^	36.7 (4.6)	0.57 (–1.41 to 0.26)		33.2 (7.3)^c^	32.6 (4.4)^d^	0.09 (–0.88 to 1.06)	
Rumination (RRQ^g^)	37.4 (7.4)^b^	35.2 (5.9)^b^	0.32 (–0.52 to 1.16)		40.1 (4.9)^c^	39.4 (4.9)^d^	0.14 (–0.83 to 1.10)	
Mindfulness skills (FFMQ-SF^h^)	75.5 (7.8)^b^	77.2 (5.9)^b^	0.24 (–1.08 to 0.60)		77.6 (3.3)^c^	75.6 (4.1)^d^	0.52 (–0.46 to 1.50)	
Self-compassion (SCS-SF^i^)	47.8 (7.6)^b^	48.9 (7.2)^b^	0.14 (–0.98 to 0.69)		53.2 (7.2)^c^	54.7 (3.0)^d^	0.24 (–1.21 to 0.73)	
Positive mental health (MHC-SF^j^)	37.5 (15.9)^b^	43.3 (15.0)	0.36 (–1.19 to 0.46)		39.3 (12.7)^c^	33.7 (9.9)^d^	0.46 (–0.52 to 1.43)	
Engagement with intervention (TWEETS^k^)	24.9 (3.0)^b^	23.4 (5.7)^b^	0.32 (–0.52 to 1.16)		25.5 (2.4)^c^	16.4 (8.4)^d^	1.53 (0.44 to 2.63)	

^a^HADS: Hospital Anxiety and Depression Scale.

^b^n=11.

^c^n=10.

^d^n=7.

^e^FCRI-SF: Fear of Cancer Recurrence Inventory-Short Form.

^f^CIS: Checklist Individual Strength.

^g^RRQ: Rumination-Reflection Questionnaire.

^h^FFMQ-SF: Five Facet Mindfulness Questionnaire: Short Form.

^i^SCS-SF: Self-Compassion Scale-Short Form.

^j^MHC-SF: Mental Health Continuum-Short Form.

^k^TWEETS: Twente Engagement with E-health Technologies Scale.

## Discussion

### Principal Findings

This nonrandomized study evaluated aspects of feasibility of group, blended and individual, unguided eMBCT for people with cancer. Overall, participants were positive about their experiences in both conditions. This supports the progression to a full-scale RCT in which the effectiveness of group, blended and individual, unguided eMBCT will be assessed.

We found that participants in both intervention conditions valued practicing at their own time, at any place. This flexibility of eMBCTs among people with cancer has been previously reported as a facilitator [14,36,37]. It is evident that many people with cancer need flexible psycho-oncological interventions.

Participants in the group, blended condition particularly valued the group component of the sessions; they felt connected with others, experienced good peer support, and appreciated the synchronicity in communication. People in the group, blended condition even indicated that there were too few group sessions and that they would have liked more. Based on these results, a fourth group session will be added to the group, blended condition in the full-scale RCT. It should be noted that, although participants’ feedback has been mentioned as relevant in the cocreation process [10] and we obviously considered it important, it should be critically evaluated and weighed. Participants in this study reported that they preferred the group sessions, but if we were not able to offer the group, blended condition, they still accepted the individual, unguided condition. It is clear that participants saw the group as an important component, yet this does not necessarily mean that the group sessions are indeed crucial for the intervention to be effective. In fact, it was reported that human feedback was the most requested feature in a participatory design for an online intervention; however, this did not increase effectiveness, and the feedback messages were not even read all the time [38]. Hence, there might be a difference in what people say they want prior to participation, what they will actually do or use, and what is effective. Therefore, it is crucial to carefully think about how to use different kinds of input in a cocreation process when developing online interventions.

In terms of preferences and dropouts, the first 12 participants who enrolled in this study preferred the group, blended condition, and there were no dropouts in this condition, compared with 5 participants who discontinued the intervention in the individual, unguided eMBCT. Acceptability and adherence seemed to be higher in the group, blended condition than in the individual, unguided condition. It is important to note that all participants who discontinued the intervention were in palliative treatment. Although we could not determine the exact reason, these preliminary insights suggest a proclivity for group, blended eMBCT and questions the acceptability of the individual, unguided eMBCT for people receiving palliative treatment. In addition, it should be considered that, although the individual, unguided condition may be easier to implement and cheaper, people with cancer may still prefer a group, blended intervention format.

Another finding is that the completion rates for the postintervention questionnaires and interviews were low, in particular for the individual, unguided group. Participants were not reminded to complete questionnaires, and no incentive to participate in the interviews was provided. In addition, people in the individual, unguided condition had no contact with a mindfulness teacher or peers throughout the intervention. It might be that these participants did not feel as engaged in the study as the people in the group, blended condition. It has been shown that the use of electronic reminders and real-time monitoring among people with cancer can contribute to a very high completion rate [39]. In the full-scale RCT, we therefore plan to include prompts, such as emails, calls, and WhatsApp messages, so participants are reminded to complete questionnaires.

### Strengths and Limitations

In this pilot study, we included representatives from the target group as well as experts in the field of cancer, mindfulness, and eHealth to develop an effective intervention. This study highlights the importance of assessing relevant stakeholders’ opinions before developing an intervention and prior to going through the efforts of conducting a full-scale RCT. Based on the results of the cocreation process, we developed an app that is visually attractive; user friendly; low cost; and flexible in how, when, and where to participate. We developed an intervention that is in line with the participants’ needs and wishes and that considered expert opinions. It has increasingly been mentioned in the emerging field of eHealth interventions [10] that it is crucial to carefully consider and understand the target group when developing an effective online intervention. In this pilot study, we not only carefully addressed the target group’s desires and needs before the intervention but also evaluated their experiences after, to develop an optimal intervention that is acceptable to the end user.

This study also has limitations that should also be considered when interpreting its results. First, because of the nature of pilot studies, this study had a small sample size; in addition, it was nonrandomized, limiting our ability to assess limited efficacy. Second, our sample was rather homogeneous (eg, all Dutch, mostly highly educated women), and these participants were self-selected. The participants who had a choice between both conditions all chose the group, blended format. Consequently, findings cannot be generalized to all people with cancer. It may require more research to be able to apply online MBIs across people with cancer with different characteristics (eg, type of cancer, age, sex, language). Moreover, some participants who were invited for the interviews did not reply, and some declined participation, which further limits the generalizability of our results and calls for further research: More attention needs to be paid to people who are not reached or who do not choose to participate. In addition, in our study, we only assessed barriers and facilitators for the interventions among those who had already agreed to participate. Gaining more in-depth knowledge about those who declined participation in the program could have provided additional information about the acceptance of the program.

### Research Implications

MBCTs have proven to be effective for people with cancer [7,11], and here, we showed that participants felt positively about the 2 formats of eMBCT. Although all interviewed participants considered the intervention conditions acceptable, there were differences in their experiences both between and within intervention conditions. Participants experienced the same components of an online intervention in different ways, which is in line with the findings of similar studies [14,37]. For instance, a study about an online MBI among people with cancer found that some participants found the meditations too long, whereas others liked how they enabled them to have time for themselves [37]. In our previous study, we also found that many aspects of the eMBCT (such as the treatment setting and format) were mentioned both as a facilitator and a barrier [13]. In this pilot study, we did not assess specific participants’ characteristics that might explain these differences. Exploring which type of program delivery works for whom can help to establish the best fit for individual patients, balancing effectiveness and the resources required. The subsequent full-scale RCT with a larger and more varied sample will enable us to conduct mediation and moderation analyses to help clarify some of these uncertainties.

It should be noted that participants valued the possibility of following the program at their own time and place. Being able to decide when and where to participate in online interventions among people with cancer has been reported as a positive characteristic among other pilot studies too [14,37,38]. In addition, to our knowledge, there are no studies comparing preferences of people with cancer between online, group, blended eMBCT and individual, unguided eMBCT. This highlights the importance of research on effectiveness among online MBIs for people with cancer.

### Conclusions

The main goal of this study was to assess aspects of feasibility of group, blended and individual, unguided eMBCT for people with cancer. This study showed that both intervention conditions were positively received and could potentially be effective. The results of this investigation inform adjustments to the intervention and study process prior to conducting a full-scale RCT to evaluate its effectiveness [18].

## Appendix

Multimedia Appendix 1 Description of the intervention components and interview guide.

## Abbreviations

CONSORT: Consolidated Standards of Reporting Trials
eMBCT: online mindfulness-based cognitive therapy
MBCT: mindfulness-based cognitive therapy
MBI: mindfulness-based intervention
RCT: randomized controlled trial

## Data Availability

The data sets generated and/or analyzed during this study are available from the corresponding author on reasonable request.
