# Supplementary material for: Group, Blended and Individual, Unguided Online Delivery of Mindfulness-Based Cognitive Therapy for People With Cancer: Feasibility Uncontrolled Trial
Source: JMIR Form Res. 2024 Feb 21;8:e52338. doi: 10.2196/52338 (PMC10918539; doi:10.2196/52338)
Supplement: Multimedia Appendix 1 [file formative_v8i1e52338_app1.docx]

# Detailed description of the intervention components in Minddistrict

Participants followed the intervention via the web-based platform Minddistrict. They could access to it at any time, from any place, at no cost, via their phones, tablet, or computer. The intervention in Minddistrict started with an introductory session explaining what the online mindfulness training entails, a tour of the online environment, and education about how mindfulness can be useful for people with cancer. Additionally, participants could choose between two avatars as the virtual mindfulness teacher (coach) who would guide them through the online sessions (see Figure 1).

The intervention in Minddistrict followed the core-components of an MBI: mindfulness exercises, psychoeducation, reflections, homework, and a silent day. The mindfulness exercises consisted of guided meditations through audio-files, or yoga exercises (see Figures 2 and 3). Psychoeducation was provided in the form of animated videos and videos from previous cancer participants who shared their experiences with MBCT (see Figure 4). Reflections were prompted by asking participants different questions about their experiences with the content of the sessions. Some of the reflections were presented as multiple-choice questions with answers that participants could choose. The answers were common responses that people with cancer had previously reported. After selecting one answer, participants received automatic feedback from the virtual mindfulness teacher (see Figure 5). Daily homework was provided after completion of each session (see Figure 6). In addition, participants had the option to fill an online logbook which served as a mindfulness diary. At the end of each session, participants could see how much they have been progressing. This was depicted with a metaphoric evolution of a seed to a tree that gave fruit, this resembled their own growth throughout the intervention (see Figure 7).

Participants could decide to complement the online program with a physical workbook that was sent via regular mail before starting the intervention. Additionally, participants could choose to include reminders to do the exercises, which were sent to their personal email. Finally, there was a help-desk available during working hours, they could contact us via phone or email for any urgent question, psychological complaint, or technical issue.


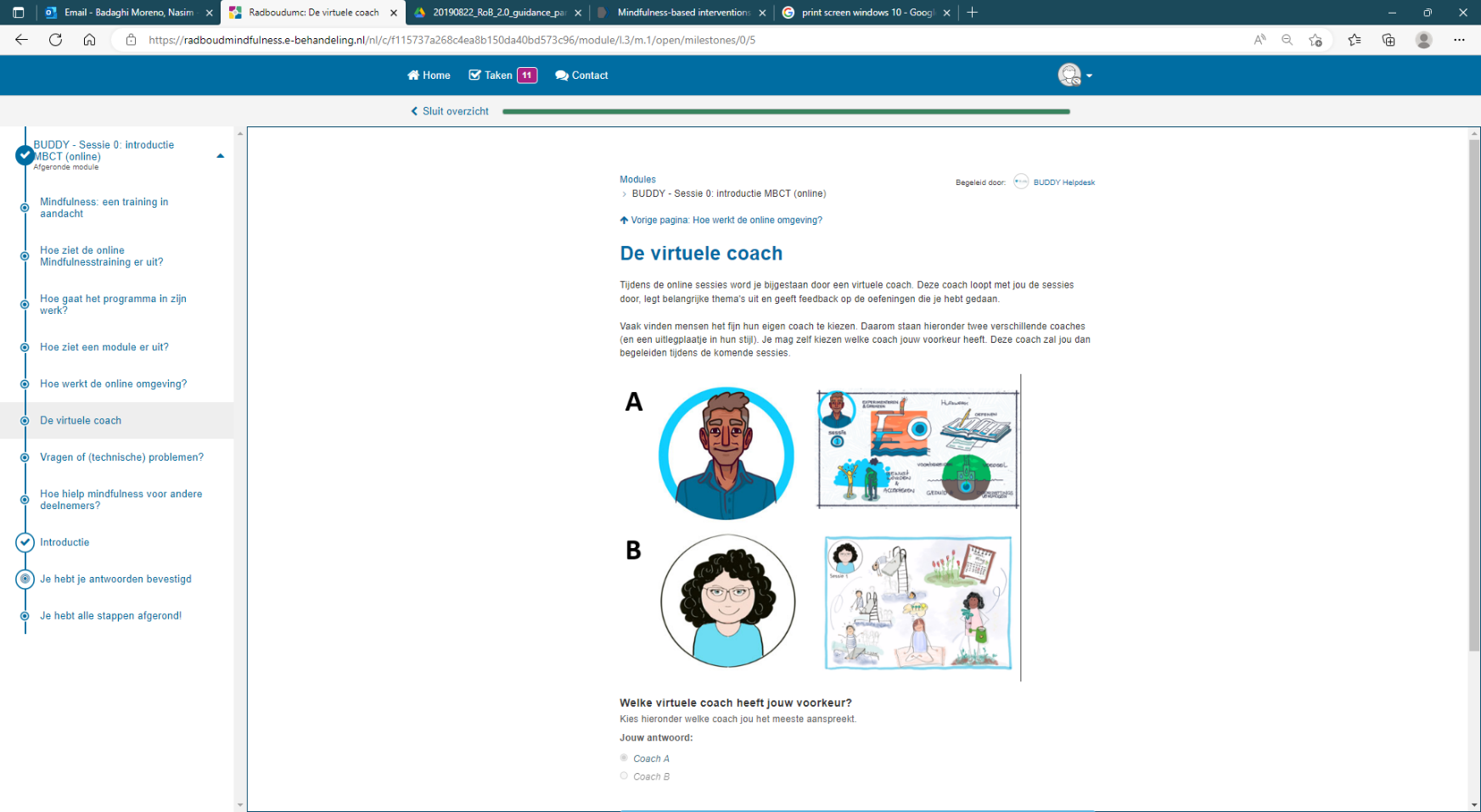
Figure 1. Virtual coach.


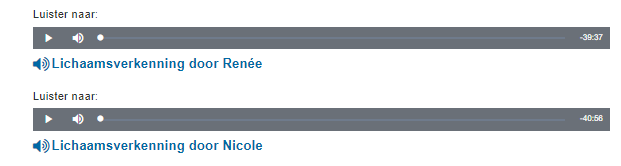
Figure 2. Meditation audio-files.

Figure 3. Yoga exercises.


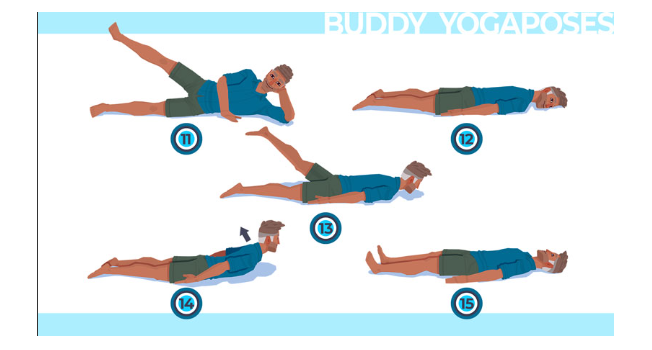


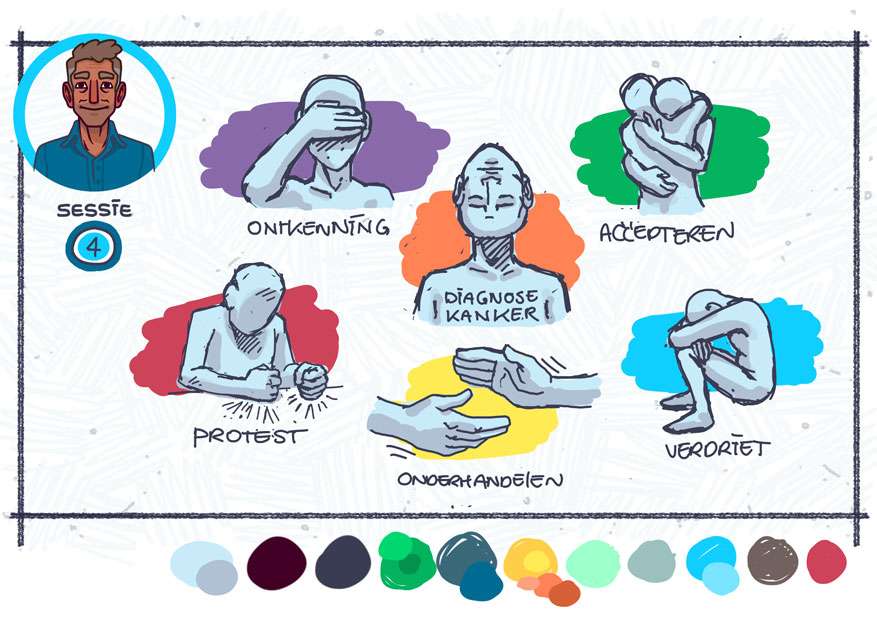
Figure 4. Animated psycho-educational videos.

Figure 5. Automatic Feedback


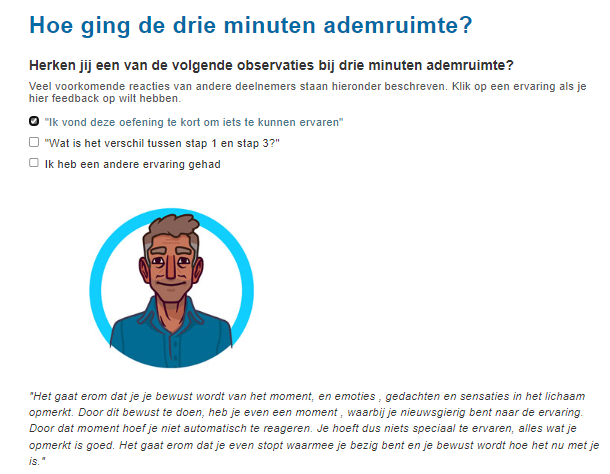


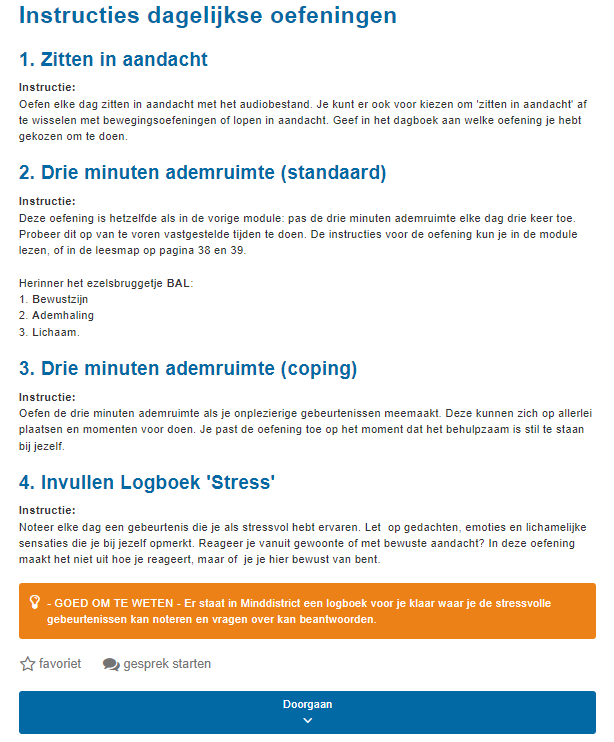
Figure 6. Homework

Figure 7. Growing tree.


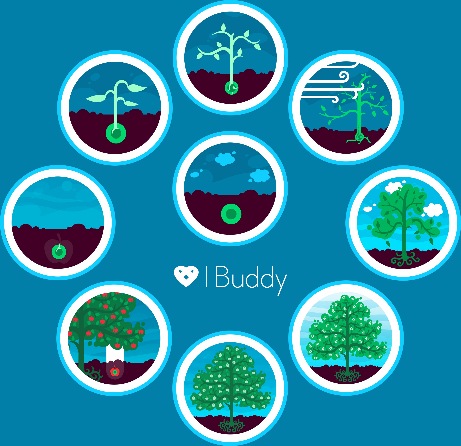


# Co-creation Process

The aim of the co-creation process was to develop and optimize the characteristics and components of the two eMBCT interventions. Both conditions shared the same core content on the platform Minddistrict. The co-creation process focused on devising appropriate content and tasks to support the individual use of the intervention platform.

The co-creation process consisted of six meetings of two hours each. In these meetings, experts in e-Health, MBCT teachers, representatives from cancer patient organizations, people with cancer (total N = 10), and four of the authors (AS, SK, MvK) exchanged ideas to identify user needs and requirements for an effective eMBCT intervention. Additionally, the stakeholders participated and evaluated a prototype of the individual unguided eMBCT intervention.

Two meetings were in-person group interviews and four were online group interviews (due to social restrictions from COVID-19). During the first meeting, the stakeholders stated the themes they found important to discuss for the next five meetings. Main themes were related to the process of the intervention (e.g., how participants expect and are expected to engage in the intervention and how the individual and group sessions in the group blended format are connected to each other), the progress participants make (e.g., if and how progress could be visualized), and the provision of feedback (e.g., if and how automated feedback could be provided). Between the meetings, the stakeholders were asked to complete one eMBCT prototype session and test all its features. Afterwards, they received a questionnaire to report their feedback. Subsequently, themes, experiences, and feedback on the prototype were discussed. These discussions provided detailed information on how to adapt text and exercises to better fit the different needs and circumstances of people with cancer. If the feedback provided by a stakeholder was widely supported and changes could be implemented, then adaptations were made; however, if the feedback provided was too personal or was to difficult to implement, then no adaptations were made. Attempts were made to integrate all feedback, but that was not always possible. The researchers made the final decisions on what (and how) to include all input.

As a result of these discussions, themes for the development of the intervention were identified, which focused on increasing participants’ motivation, adherence, and engagement to the intervention. The themes were: personalisation, automated feedback, depicting progression during the intervention, increasing motivation to participate in the intervention, and facilitation of the silent day. The following aspects were implemented in the final version of the intervention for this study: *personalisation,* the possibility to choose a virtual coach with a certain style that would be used throughout the intervention; *‘automated feedback,* preformulated feedback generated automatically as a response to possible questions about certain exercises within the intervention; *depicting progress,* a picture was added at the start of each intervention session illustrating the progress a participant made through the program; *increasing motivation,* reminders, positive feedback, and videos from previous participants who share their experience with MBCT were included*; facilitation of the silent day*, clear and structured guidance in the app, videos from previous participants sharing their experiences about having following a silent day. Minddistrict was the web-based platform used for both eMBCT conditions, meaning that participants found the necessary information to follow the intervention in Minddistrict. A detailed description of the online intervention components and screenshots can be found in the supplementary files.

# Interview Guide Qualitative Interviews Buddy

1. Introduction:

- Explain research, goal of the research, and aims from this interview: participants' experiences with following the respective intervention condition.
- State approximate duration of the interview, no wrong answers, and explain confidentiality.
- Ask if everything is clear or if there are any questions.
- Start recording. Say the date, participation number, and permission to record.

1. Main questions:

- What in the online format helped you to follow the training?
- Were there specific parts that were helpful?
- What in the online format was not helpful for you in following the training?
- Were there specific parts that were not helpful?
- What would you like to change about the training?
- Does the (…) evoke something from you?
- Ask about each component: virtual coach, automatic feedback, infographics and sound files, videos from the participants, silent day, reflections and diaries, Minddistrict environment.

*Ask for clarification and elaboration for each question when answers are too general. For each question ask what the participant found helpful/unhelpful. Be open during the interview and (when pertinent) follow-up with questions based on what participants say.*

1. Closing:

- Are there things that you have not been able to share yet or that we have not discussed?
- Is there anything you would like to emphasize or to add?
- Mention that we can always make another appointment later if something comes up.
